# Flexible Ag Microparticle/MXene-Based Film for Energy Harvesting

**DOI:** 10.1007/s40820-021-00729-w

**Published:** 2021-09-24

**Authors:** Yunpeng Jia, Yamin Pan, Chunfeng Wang, Chuntai Liu, Changyu Shen, Caofeng Pan, Zhanhu Guo, Xianhu Liu

**Affiliations:** 1 College of Materials Science and Engineering, Key Laboratory of Advanced Material Processing & Mold (Ministry of Education), National Engineering Research Center for Advanced Polymer Processing Technology, Zhengzhou University, Zhengzhou, 450002 People’s Republic of China; 2 National Center for Nanoscience and Technology (NCNST), Beijing Institute of Nanoenergy and Nanosystems, Chinese Academy of Sciences, Beijing, 100083 People’s Republic of China; 3 College of Physics and Optoelectronic Engineering, Shenzhen University, Shenzhen, 518060 People’s Republic of China; 4 Integrated Composites Laboratory, Department of Chemical & Biomolecular Engineering, University of Tennessee, Knoxville, TN 37996 USA

**Keywords:** Energy harvesting, MXene, Ag microparticle, Triboelectric nanogenerator

## Abstract

Ultra-thin flexible films have attracted wide attention because of their excellent ductility and potential versatility. In particular, the energy-harvesting films (EHFs) have become a research hotspot because of the indispensability of power source in various devices. However, the design and fabrication of such films that can capture or transform different types of energy from environments for multiple usages remains a challenge. Herein, the multifunctional flexible EHFs with effective electro-/photo-thermal abilities are proposed by successive spraying Ag microparticles and MXene suspension between on waterborne polyurethane films, supplemented by a hot-pressing. The optimal coherent film exhibits a high electrical conductivity (1.17×10^4^ S m^−1^), excellent Joule heating performance (121.3 °C) at 2 V, and outstanding photo-thermal performance (66.2 °C within 70 s under 100 mW cm^−1^). In addition, the EHFs-based single-electrode triboelectric nanogenerators (TENG) give short-circuit transferred charge of 38.9 nC, open circuit voltage of 114.7 V, and short circuit current of 0.82 μA. More interestingly, the output voltage of TENG can be further increased via constructing the double triboelectrification layers. The comprehensive ability for harvesting various energies of the EHFs promises their potential to satisfy the corresponding requirements.

**Supplementary Information:**

The online version contains supplementary material available at 10.1007/s40820-021-00729-w.

## Highlights

- Flexible Ag microparticle/MXene-based energy-harvesting films were fabricated via simple spraying and hot-pressing.
- Optimal film shows high conductivity and effective electro/photo-thermal abilities.
- It can be easy assembled single-electrode TENG and the output voltage is enhanced by assembled two triboelectrification layers.

**Supplementary Information:**

The online version contains supplementary material available at 10.1007/s40820-021-00729-w.

## Graphical abstract

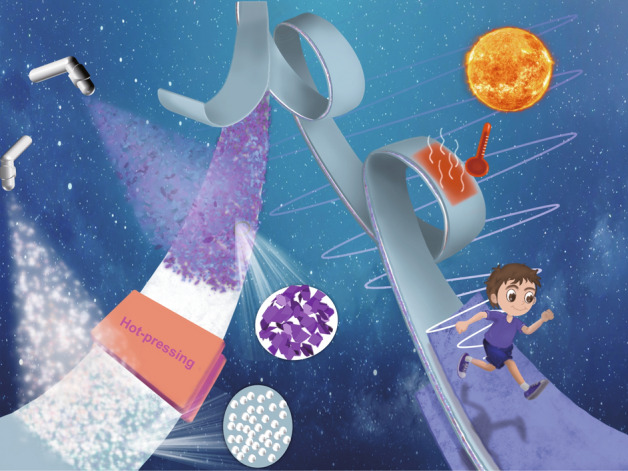

## Introduction

The consumption of natural resources such as fossil fuels continues to increase with the rapid development of the economy, and we are facing increasingly serious energy shortages and environmental damage. The efficient utilization of renewable resources (e.g., solar, wind, and tidal power) has been extensively explored; meanwhile, biomechanical energy from human movements is considered as an emerging way to provide the driving force for equipment [1–3]. In this context, the research of developing energy collection devices for ensuring energy security, realizing recycling, and promoting sustainable economic development is of great significance.

Energy-harvesting films (EHFs) are considered to be ideal candidate in this respect, which have been widely applied in solar cells [4–6], light-emitting components [7, 8], heating devices [9, 10], and so on. In general, the current EHFs collect various forms of energy by introducing fillers with different properties including carbon nanotubes, metal nanowires and conducting polymer to the elastic matrix [11–17]. Nevertheless, the nanofillers are easy to agglomerate during the preparation process and limit the application in practice [18, 19]. Therefore, it is extremely important to select fillers with good dispersion and excellent performance. In particular, a newly emerged two-dimensional (2D) transition metal carbonitride noted as MXene, has shown surprising properties in the fields of electrical energy transmission and visible light absorption. The multilayer 2D material possesses high electrical conductivity (~ 10^4^ S cm^−1^), as well as a large specific surface area and excellent dispersion, making it an ideal candidate for fabricating appreciable EHFs [20–23].

Triboelectric nanogenerator (TENG), converting mechanical energy, especially the human activities, into electricity by coupling the electrification and electrostatic induction, enables the energy storage and self-powered signal collection [24–26] and therefore has been highlighted combined with their portability, durability, material diversity for triboelectric layer (e.g., flexible polymers) and electrode (e.g., conductive hydrogel or fabric), and cost-effectiveness. The triboelectric layer usually has a strong triboelectrification effect and is less conductive or insulating, which can capture the transferred charges and retain them for an extended period of time, to build up the electrostatic charges and potential difference [27]. For example, polyurethane has been selected as a triboelectric layer more frequently due to its strong electronegativity, excellent flexibility, and environmental friendliness [28, 29]. In another aspect, the electrode with a conductive network enables the electrons’ transportation and the charging and discharging process. In particular, a hybrid electrode layer with the embedded conductive fillers can synergistically trap and block the charges, thereby further improving the electrical output capacity [30–33]. Through the constant contact-separation mode, TENG could convert mechanical energy into electricity by generating an objective electric current, enabling the construction of the self-powered systems.

Herein, the multifunctional EHFs were developed by spraying Ag microparticles (AgMPs) and MXene dispersed solution in sequence between on waterborne polyurethane (WPU) layers, featuring remarkable mechanical properties and high electrical conductivity. On the one hand, the EHFs can convert electrical energy and light energy into heat with a maximum of 121.3 and 66.2 °C, respectively, indicating the reliability in application of electric-photo-thermal. On the other hand, electrons can be quickly transferred in the EHFs benefiting from the low resistance of the filler layer, and thus the composite films can be assembled into a single-electrode TENG (STENG) assisted with the strong triboelectric effect of the WPU layer. The EHFs with stable electrical output can provide power for electronic components, and their outstanding waterproof ability also broadens the application range. This work confirmed that the EHFs possess the feasibility of in-depth exploration in energy collection, utilization, and transformation.

## Experimental Section

### Materials

Lithium fluoride (LiF, 99%) was purchased from Aladdin Reagent Co., Ltd. Ti_3_AlC_2_ powder (MAX, 400 mesh) was obtained from Jilin 11 Technology Co., Ltd. Ag microparticles (NO-M-004–3) with a size of 1 μm were purchased from Shanghai Naiou Nano technology Co., Ltd., with a purity of ~ 99.9%. And commercial WPU was supplied by Guangzhou Dolphin New Materials Co., Ltd., with a concentration of 50 wt%. Besides, Acrylic sheets and silicone rubber were purchased from Shanghai Yu Zhao Industrial Co., Ltd.

### Synthesis of Ti_3_C_2_T_x_ MXene Nanosheets

As previous research has stated, Ti_3_C_2_T_x_ MXene nanosheets were obtained by etching Ti_3_AlC_2_ in hydrofluoric acid (HF). Firstly, LiF (2 g) was slowly introduced into a Teflon beaker containing 40 mL HCl (9 M) with stirring continuously at 35 °C for 30 min. Then, at an extremely slow pace, 2 g Ti_3_AlC_2_ was added to it. The etching process, which takes 24 h under magnetic agitation, aims to remove the aluminum layer. After it, the solution was centrifuged at 3500 rpm for 10 min to collect the precipitate. The precipitate was further treated by ultrasound and centrifugation until reaching a suitable pH value (about 6). Then, the multilayered Ti_3_C_2_T_x_ MXene was dispersed in ethanol and ultrasonicated for 1 h. After centrifugation at 10,000 rpm for 10 min, the obtained products were further treated by ultrasound to remove the unexfoliated Ti_3_C_2_T_x_ and collected. Finally, the 2D Ti_3_C_2_T_x_ MXene nanosheets were obtained by freeze-drying at -80 °C for 3 days.

### Fabrication of EHFs

**Fig. 1:**
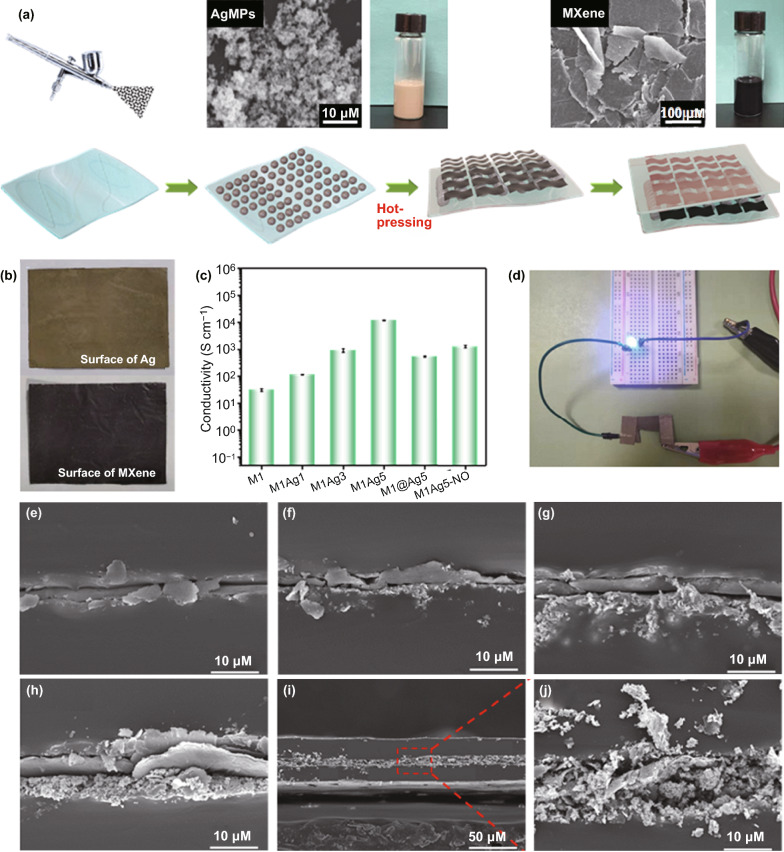
**a** Preparation process of the EHFs. The illustrations are SEM images of AgMPs and MXene sheets as well as their dispersed solutions. **b** Digital photographs of both sides of EHFs. **c** Electrical conductivity of EHFs. **d** Digital photographs of lighting up LED by a power supply. Cross-sectional SEM images of **e** M1, **f** M1Ag1, **g** M1Ag3, **h** M1Ag5 and **i-j** M1@Ag5 EHFs

Following the schematic exhibited in Fig. 1, the multilayered WPU/AgMPs/MXene/WPU films were fabricated by a layer-by-layer spraying process. Typically, different amounts of AgMPs or MXene were dispersed in an equal mixture of alcohol and water assisted by a hot plate to shorten the evaporation time during the spraying process. Then, the corresponding suspensions were evenly sprayed onto the Teflon substrate with a commercial spray gun at a distance of 10 cm and a pressure of 2 bar, respectively. Firstly, AgMPs are sprayed on the first layer of WPU, after it, the film is then transferred to a vacuum press (100 °C, 1 MPa) for 20 min to make the metal particles hot sintering process. Then, the MXene dispersion was coated on the sintered layer to form the composite films. Finally, the second layer of WPU is overlaid on it for coating. Ag layer, with different areal densities (1, 3, and 5 mg cm^−2^), was used as the core layer along with the MXene layer.

### Fabrication of Sandwich-Structured STENG

The preparation process of STENG is simple and efficient. Before spraying the MXene layer, a copper wire was introduced, and then the resulting composite film was affixed to the acrylic substrate with double-sided tape. What needs to be explained is that the top WPU layer acts as a positive dielectric material and the MXene and Ag layer act as a synergistic electrode.

### Characterization

The morphology of the MxAgy films was characterized by field emission scanning electron microscopic (FE-SEM, JSM-7001F) at an accelerating voltage of 20 kV. The volume conductivity of samples was performed with the aid of a digital multimeter (Tektronix DMM4050), and the value can be calculated by the following equation: $\sigma = L/{\text{RS}}$, where $\sigma$ is the volume conductivity, $L$ is the length of both ends, $R$ is the volume resistance, and $S$ is the cross section area of the film. The transmittance spectrum was measured by UV–Vis NIR absorption spectrometer (Cary 5000) with the measured wavelength range of 300–2400 nm.

A universal test platform (UTM2203, Shenzhen Suns Technology Stock Co., Ltd., China) controlled by a custom computer was used and equipped with a 100 N force sensor to measure mechanical properties. The stress–strain curves of MxAgy films were carried out under uniaxial test conditions with a crosshead speed of 5 mm min^−1^.

The electro-photo-thermal property was characterized by an infrared (IR) thermal imaging instrument (FLIR, E60), and the temperature change was real-time-recorded. The difference is that the electro-thermal test was done by applying a voltage to the sample, while the photo-thermal test was done by exposing the membrane to a solar simulator. The value of Q_SC_, V_OC_, and I_SC_ was precisely recorded by an electrometer (Keithley-6514). It was carried out under the frequency-controlled force generated by the motor.

## Results and Discussion

### Fabrication and Structure of the MxAgy EHFs

The schematic process of multifunctional EHFs is illustrated in Fig. 1a. Briefly, on one side of the prepared WPU film, AgMPs are sprayed and then subjected to a certain condition of hot-pressing treatment. The purpose of the hot-pressing process is mainly for sintering AgMPs. The activation energy required for surface diffusion is relatively low, and the high temperature can just provide the required energy. Furthermore, the surface diffusion has a certain effect on the formation and expansion of the sintering neck, and the necks between the particles become wider under high-pressure conditions to form an effective metallic bonding [34, 35]. As shown in Fig. S1, before sintering, the conductivity of the Ag layer is relatively low, which is due to a sparse percolation network of particles. During the hot-pressing process, AgMPs are transformed into a coherent microstructure at an elevated temperature, and subsequently, the ligament size of nano-porous Ag increases, leading to the complete bonding of AgMPs with each other (Fig. S2). After hot-pressing, MXene dispersion and WPU emulsion were sprayed sequentially. The corresponding composite films are described as MxAgy, where x and y represent the areal density (mg cm^−2^) of MXene and AgMPs, respectively. In addition, a mixed MXene and AgMPs was prepared, which is named M1@Ag5 for comparison. M1Ag5-NO represents the sample without the hot-pressing treatment of AgMPs. The two sides of EHFs have different colors (Fig. 1b), and the electrical conductivity of EHFs increased as the AgMPs contents increased (Fig. 1c), enabling the lighting-up of a LED under a power supply (Fig. 1d).

The scanning electron microscopy (SEM) images of films show a typical layer-by-layer structure (Fig. 1e–j). In Fig. 1e, it can be clearly seen that MXene sheets are sandwiched between WPU layers. With the introduction of AgMPs, an obvious granular layer appeared below MXene. It is worth noting that due to the effect of the hot-pressing, some AgMPs will be pressed into the bottom WPU layer, which may enhance the mechanical properties of the film for the embedding of fillers (Fig. S3). More importantly, no obvious interface defects are discovered between these layers, which is conducive to ensure performance stability in different conditions. In contrast to the mixed-phase film of M1@Ag5, AgMPs are randomly distributed on the MXene layer, and the overall structure was relatively loose. Moreover, the thin thickness of MxAgy (~ 50 μm) and conductive layer (~ 10 μm) make it great potential in miniaturized and lightweight regions.

### Electric-Thermal and Photo-Thermal Performances

**Fig. 2:**
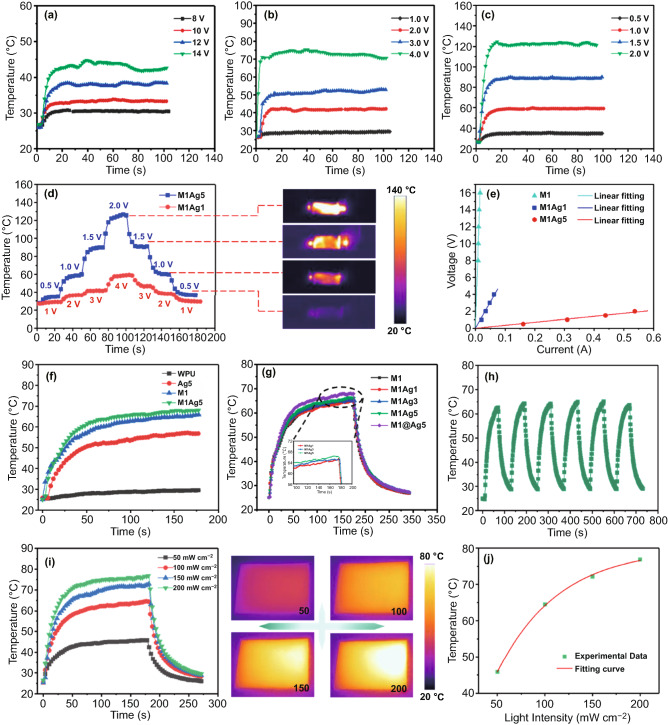
Temperature variation at different applied voltages of **a** M1, **b** M1Ag1, **c** M1Ag5 EHFs. **d** Temperature evolution of M1Ag1 and M1Ag5 upon gradient changed voltages. **e** Voltage–current curves. **f, g** Temperature of different films under 100 mW cm^−2^. **h** Temperature variation curve under cycles of on−off light illumination. **i** Temperature difference of M1A5 film under different irradiated power densities. **j** Experimental data and fitting curve of peak temperature versus the irradiated power density

According to the principle of Joule heating, heat can be generated when electric currents pass through a conductor. Joule's law can be expressed by Eq. 1:

1 $$Q = UIt = I^{2} Rt = \frac{{U^{2} }}{R}t$$

where $Q$, $U$,$I$, $R,$ and $t$ represent generated Joule, applied voltage, current, resistance, and operating time, respectively [36, 37]. For the convenience of research, all the tests are characterized in the surface of AgMPs. Figure 2a–c reflects the timely feedback of Joule-heating temperature of M1, M1Ag1, M1Ag5 under different voltages. The temperature of M1Ag1 and M1Ag5 can rapidly heat up to the platform of 72.1 and 121.3 °C within 20 s, with applied voltages of 4 and 2 V, respectively. However, the control group M1 can only heat up to about 43.6 °C, even at a high voltage of 14 V, ascribing to the low conductivity of M1, as depicted in Fig. 1c. The M1Ag5 can maintain a stable temperature platform under the long period of working conditions (Fig. S4). By applying different gradients of external voltage, the EHFs exhibit controllable cycle stability and temperature responsiveness (Fig. 2d). During the energization process, the temperature distribution on the film surface is uniform, and the brightness of the infrared image obviously increases as the voltage rises. Moreover, the stability of the heater is also an important indicator of whether it can work for a long term. As shown in Fig. 2e, the voltage–current curves of all three samples are described, indicating that the constant resistance of the composite films and the height conforms to Ohm's law through the fitting curves under different voltages. While, due to the polymer matrix, the heating upper limit of the heater is subject to certain restrictions.

As shown in Fig. 2f, contrast to the pure WPU film, the EHFs of M1, Ag5, and M1Ag5 exhibit excellent photo-thermal abilities, which can be heated to 54.2, 64.3, and 66.2 °C, respectively, within 70 s when exposed to 1 sun irradiation (100 mW cm^−2^), ascribing to the effective photo-thermal effect of AgMPs and MXene. The higher overall temperature of the MxAgy films compared to M1 and Ag5 confirms the synergetic photo-thermal effect of MXene and AgMPs (Fig. 2g, a clearer photograph is shown in Fig. S5), for the coherent oscillation of the surface electrons induced and localized surface plasmon resonance (LSPR) of metal particles by light [38, 39]. Similarly, the semi-metallic properties of MXene also exhibit this similar property [40, 41]. When AgMPs of 1 mg cm^−2^ are sprayed, the photo-thermal effect of the EHFs is hardly improved. What’s more, it can be concluded that the hybrid EHFs possess a higher absorbance in the visible wavelength range of 400 to 800 nm (Fig. S6), which provides the most important factor for the enhancement of the photo-thermal effect. Figure 2h demonstrates that the photo-thermal performance of the film is stable under the cyclic irradiation of 100 mW cm^−2^, and the photo-to-heat output capacity does not decrease obviously. Furthermore, the temperature of the M1Ag5 film rises from 42.3 to 76.5 °C with the increase in light irradiation intensity from 50 to 200 mW cm^−2^, and the infrared image on the right visually reflects this difference (Fig. 2i). It is worth noting that the photo-thermal output of the film does not show a linear relationship with the illumination intensity (Fig. 2j), which may be caused by the inherent property of the high thermal conductivity of the AgMPs. Figure S7 reveals that with the increase in AgMPs content, the thermal conductivity in the vertical direction of the MxAgy EHFs gradually increases.

### Electrical Output Performance of the EHFs-based STENG

**Fig. 3:**
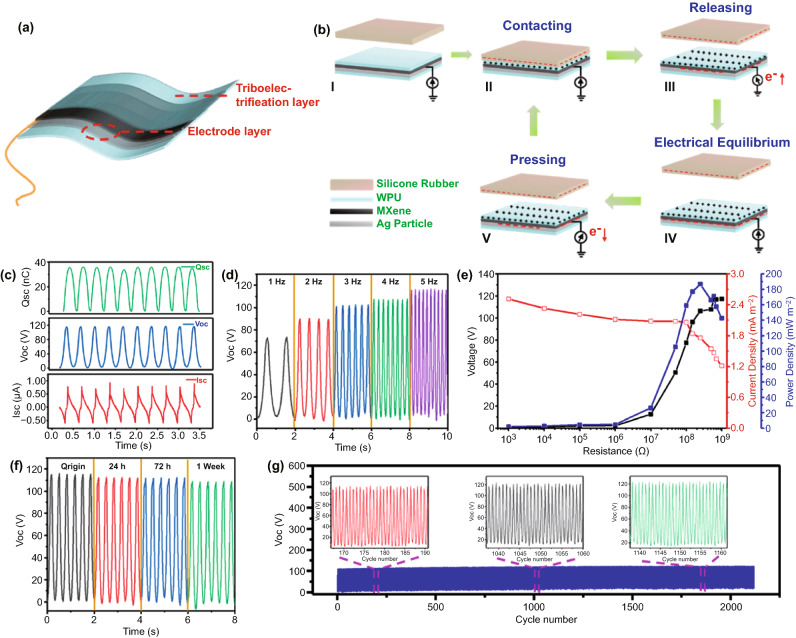
**a** EHFs-STENG and **b** its working mechanism diagram. **c** The corresponding V_OC_, I_SC_, and Q_SC_ of the STENG. **d** V_OC_ of the STENG with various frequencies (fixed pressure 10 N). **e** The output performance under the resistance of 1 kΩ–1GΩ. **f** V_OC_ of STENG in a wet environment within one week. **g** Long-term stability test of the STENG

In addition to the excellent flexibility of WPU [42], WPU exhibits a remarkable ability to gain or lose electrons when in contact with different materials as well, allowing it to be the triboelectric layer in STENG [43]. In this work, the STENG can be prepared easily by introducing a copper wire before spraying MXene (Fig. 3a). Figure 3b systematically illustrates the structure and working principle of the STENG. At first, the silicone rubber and WPU layer is determined as the negative and positive triboelectric materials, respectively, due to their difference in relative polarity (I). When the two subjects contact, negative charges will produce on silicon rubber due to its strong negative charge capturing ability, and the same amount of positive charge is generated on the surface of the WPU layer to keep the potential in balance (II). When the silicone rubber separates from the WPU layer, the unscreened positive charges will induce the accumulation of negative charges in the AgMPs/MXene interface. Meanwhile, the instantaneous electrons will flow from the ground to the AgMPs/MXene layer (III). When the separation degree between the rubber and the WPU layer reaches the maximum, the potential in the STENG system will reach equilibrium and the flow of free electrons will also terminate (IV). As rubber and WPU approach again, the electrons will flow from the MxAgy conductive layer to the ground, indicating a reversed operation mechanism (V). By repeating the contact-separation procedure mentioned above, an alternating electrical signal can be generated. The changes of the induced voltage of rubber and WPU layer during the movement process can be intuitively illustrated through the simulation of COMSOL (Fig. S8).

The electrical output performance of STENG is affected by different factors such as pressure, frequency, and contact material [44–48]. Therefore, a linear motor is used to study the property of EHFs-based STENG in the periodic contact-separation motions. The contact area is chosen as 40 × 40 mm^2^ and the tapping force is 10 N (frequency: 5 Hz). As shown in Fig. 3c, the transferred short-circuit charge (Q_SC_), the open-circuit voltage (V_OC_) and the short-circuit current (I_SC_) of the STENG are 38.9 nC, 114.7 V and 0.82 µA, respectively. The variation of output performance dependence on different AgMPs content is shown in Fig. S9. Increasing output V_OC_ and I_SC_ performance of STENG is due to the following reasons: the outstanding electrical conductivity of AgMPs causes the decrease in surface resistance significantly; the high AgMPs content leads to more effective contact area with the WPU layer. The V_OC_ of STENG at different frequencies is also studied (Fig. 3d). The V_OC_ increases accordingly with the motor frequency, which is mainly because the higher the frequency, the smaller the neutralization of positive and negative charges in the dielectric layer and the triboelectrification layer during contact, resulting in more charges gathering on the electrode and high electrical output [45]. In addition, the influence of tapping force on STENG electrical output capacity can be seen in Fig. S10. With the augment of applying pressure, the effective triboelectric area increases due to the deformation of the polymer materials [27, 49]. Figure 3e displays the output voltage, current density, and power density of the STENG by connecting various external resistors of 1 kΩ to 1 GΩ. As shown in the chart, following Ohm’s law, the voltage of the external resistance rises while the current decreases with the resistance increasing. The power density ($P$) can be calculated by Eq. 2:

2 $$P = \frac{{U^{2} }}{RA} = \frac{UI}{A}$$

where $U$ and $I$ represent the output voltage and current of the STENG, respectively. $A$ is the contact area, $R$ stands for the magnitude of the resistance. When the external load resistance is about 250 MΩ, the output power density of EHFs-based STENG can reach a maximum of approximately 186.8 mW m^−2^. The repeatability of electrical output is crucial for STENG equipment, herein, the STENG still maintains satisfactory stable outputs after over 2000 pressing-releasing circulations (Fig. 3g), showing the reliability of the device in practical application. Besides, the waterproof performance of STENG was characterized. Surprisingly, the V_OC_ remained at a similar level even after soaking in deionized water for 1 week due to the excellent waterproof performance of WPU (Fig. 3f). Additionally, the EHFs-based STENG also has a good responsiveness to human skin. As shown in Fig. S10 and Movie S1, the STENG can generate identifiable voltage signals by finger tapping and regular steps with different frequencies. This work provides a promising exploration of EHFs-based STENG in the field of human body sensors.

**Fig. 4:**
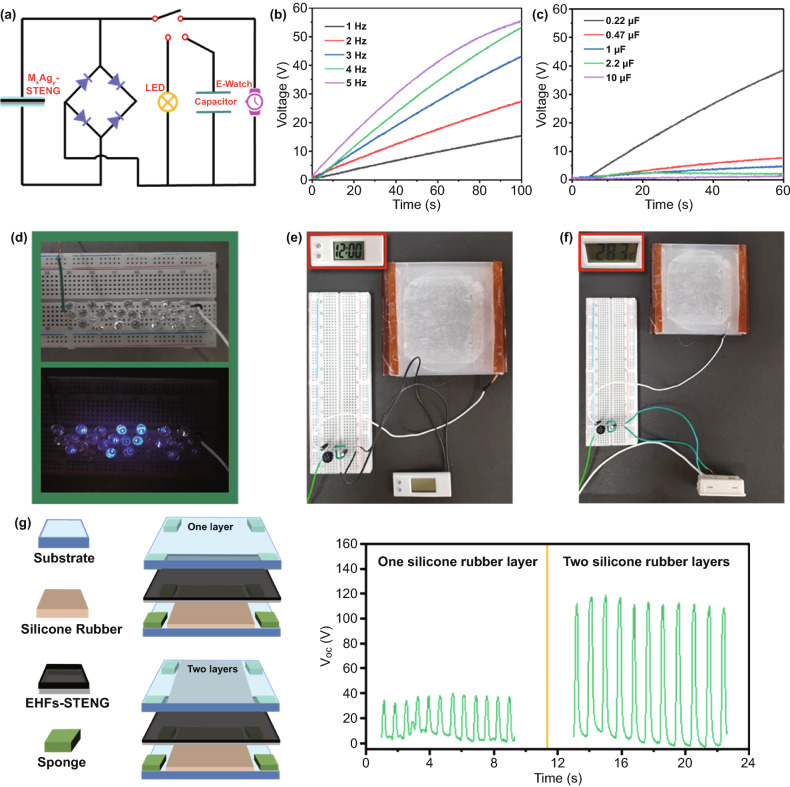
**a** Self-charging power supply system based on the equivalent circuit diagram. The charging ability of the EHFs-based STENG **b** with various frequencies (1–5 Hz) for charging a 0.22 µF commercial capacitor and **c** charging different capacitors (0.22–10 µF) at a frequency of 5 Hz. The electricity output from the STENG can drive **d** LEDs to shine, **e** electronic watches to run, and **f** electronic thermometers to work. **g** V_OC_ of the EHFs-based STENG under single and double triboelectrification layers

A self-powered system is assembled to verify the feasibility of EHFs-based STENG in practical application. As depicted in Fig. 4a, the STENG was linked to capacitor, LEDs, and electronic watch through a rectified circuit that converts the output alternating current to direct current. The charging capability of the STENG was measured by charging a 0.22 µF commercial capacitor with different operating frequencies (1–5 Hz) and capacitors of various capacity sizes (0.22–10 µF) at a frequency of 5 Hz (Fig. 4b, c). It exhibits that the charging rate increases significantly with the increasing motor frequency, which is mainly attributed to the higher contact-separation frequency of the triboelectrically layer, leading to the more accumulation of the friction charge. And, as the capacitance increases, the charging rate of the capacitor slows down reasonably. Moreover, the STENG can impel more than 20 commercial LEDs to light up by periodically clapping hands at a certain frequency (Fig. 4d and Movie S2). Further, combining with an energy storage unit, the STENG can provide driving power for portable electronic devices, such as electronic watches (Fig. 4e), electronic thermometers (Fig. 4f), etc. These results confirm that the EHFs-based STENG has the potential to be further explored in self-charging power supply system [50]. Particularly, the structure of the double triboelectrification layers is constructed to greatly increase the output voltage of STENG (Fig. 4g), which provides a potential direction for future research. The possible mechanism is shown in Fig. S12. The polarization and accumulation of charge occurred simultaneously in the upper and lower layers of WPU, and the design of double triboelectrification layers shorts the distance and speeds up the contact efficiency. The larger contact area, the higher contact-separation frequency, and the more significant the potential difference formed.

## Conclusions

In summary, EHFs were fabricated via the technology of layer-by-layer spraying and hot-pressing. The cross-section SEM images reveal the effective establishment of a stratified structure in EHFs. As expected, the high electrical conductivity and absorbance of the network constructed by the fillers endow the EHFs with low-voltage-driven Joule heating effect and remarkable photo-thermal property, respectively. Furthermore, due to the WPU layer is easy to gain or lose electrons when contacting different polarities materials, the EHFs-based STENG is verified to be able to effectively convert mechanical energy into electrical energy output and provide power for microelectronic devices in continuous contact-separation mode. This work illustrates the great potential applications of EHFs for energy harvesting to achieve better energy recycling.

## Supplementary Information

Below is the link to the electronic supplementary material.

Supplementary file1 (PDF 749 kb)

Supplementary file2 (MP4 496 kb)

Supplementary file3 (MP4 3009 kb)
