# Supplementary material for: Flexible Ag Microparticle/MXene-Based Film for Energy Harvesting
Source: Nanomicro Lett. 2021 Sep 24;13:201. doi: 10.1007/s40820-021-00729-w (PMC8463646; doi:10.1007/s40820-021-00729-w)
Supplement: Supplementary file 1 — Supplementary file1 (PDF 749 kb) [file 40820_2021_729_MOESM1_ESM.pdf]

Supporting Information for

Flexible Ag Microparticle/MXene Based Film for Energy-Harvesting

Yunpeng Jia[1, #], Yamin Pan[1, #], Chunfeng Wang[2, 3], Chuntai Liu[1], Changyu Shen[1], Caofeng Pan[2], Zhanhu Guo[4], Xianhu Liu[1, *]

[1] College of Materials Science and Engineering, National Engineering Research Center for Advanced Polymer Processing Technology, Key Laboratory of Advanced Material Processing & Mold (Ministry of Education), Zhengzhou University, Zhengzhou, 450002, P. R. China

[2] Beijing Institute of Nanoenergy and Nanosystems, Chinese Academy of Sciences, National Center for Nanoscience and Technology (NCNST), Beijing, 100083, P. R. China

[3] College of Physics and Optoelectronic Engineering, Shenzhen University, Shenzhen 518060, P. R. China

[4] Integrated Composites Laboratory (ICL), Department of Chemical & Biomolecular Engineering, University of Tennessee, Knoxville, TN 37996 USA

[#] Yunpeng Jia and Yamin Pan contributed equally to this work

*Corresponding author. E-mail: xianhu.liu@zzu.edu.cn (Xianhu Liu)

Supplementary Figures

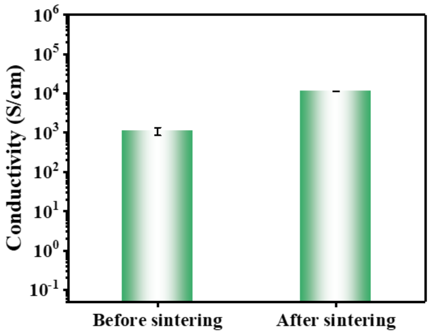

Fig. S1 Comparison of electrical conductivity before and after hot-pressing

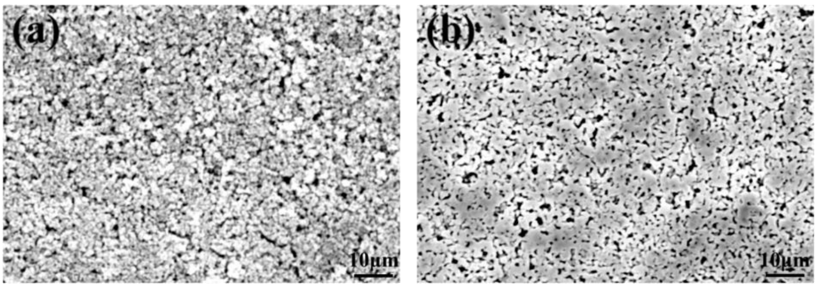

Fig. S2 Micromorphology of AgMPs **a** before and **b** after hot-pressing

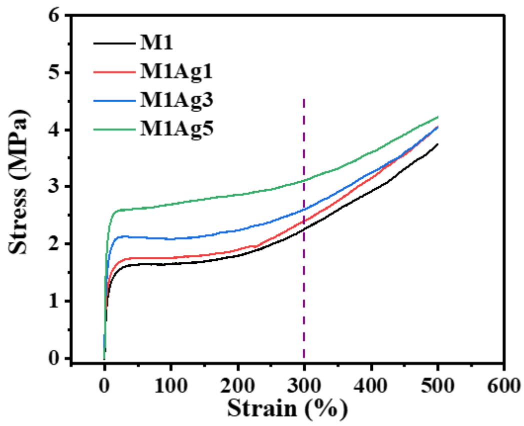

Fig. S3 Typical stress–strain curves of the MxAgy EHFs. The tensile strength increases due to the increase of AgMPs content and its embedding

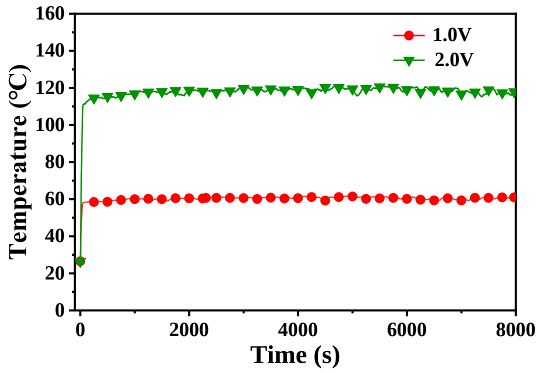

Fig. S4 Electric-thermal property of M1Ag5 film at high and low temperatures for over two hours

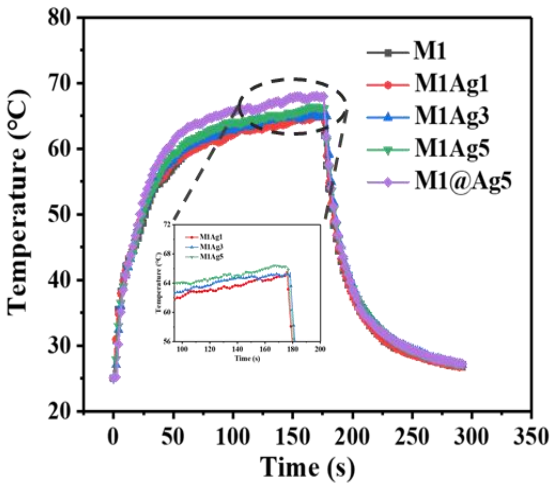

Fig. S5 Temperature of different films under 100 mW cm^{-2}

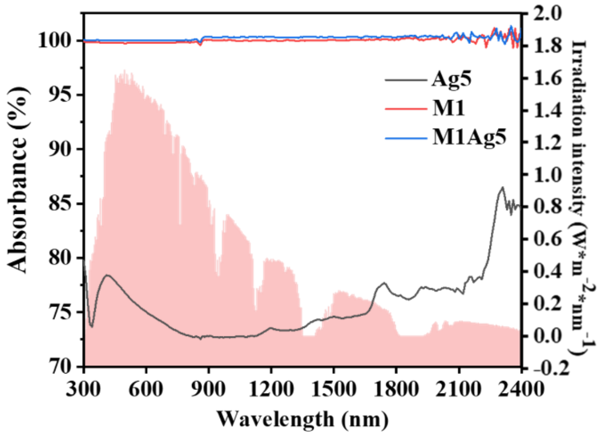

Fig. S6 Absorbance of the Ag5, M1, and M1Ag5 films

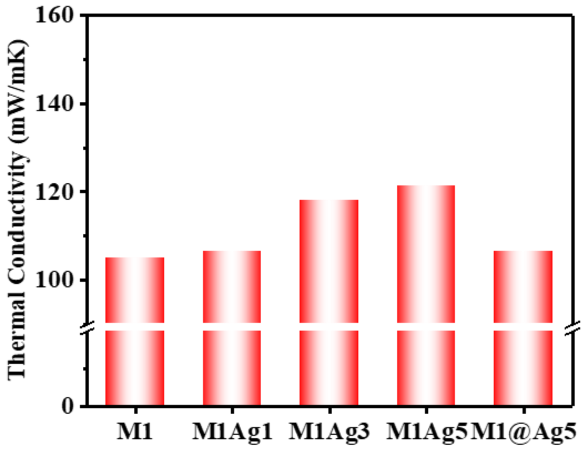

Fig. S7 Thermal conductivity of MxAgy EHFs in through-plane

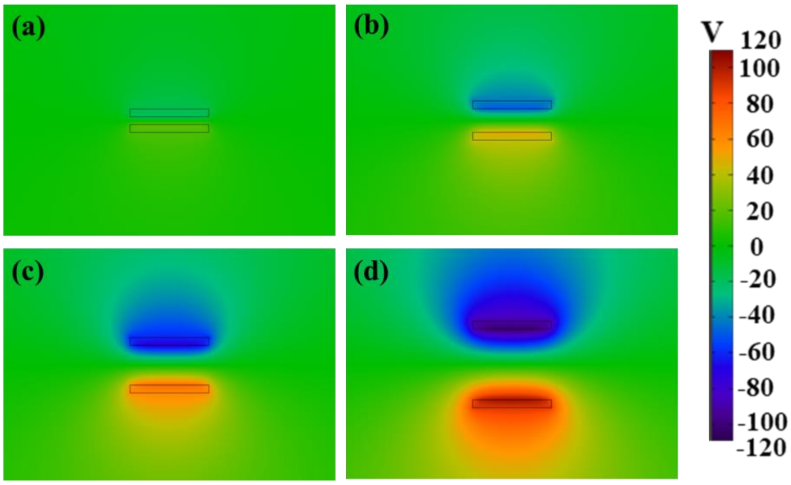

Fig. S8 Electrical potential distributions of the MxAgy-based STENG simulated by the COMSOL software

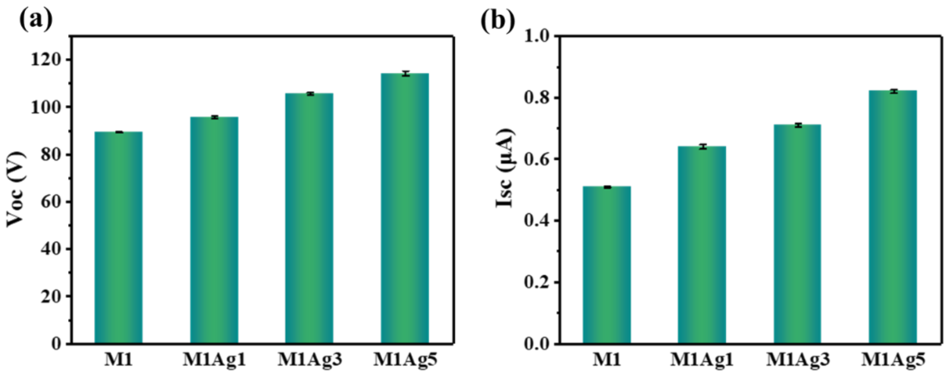

Fig. S9 Variations in the **a** V_{OC} and **b** I_{SC} for MxAgy-based STENG with various AgMPs content at a frequency of 5 Hz and a force of 10 N

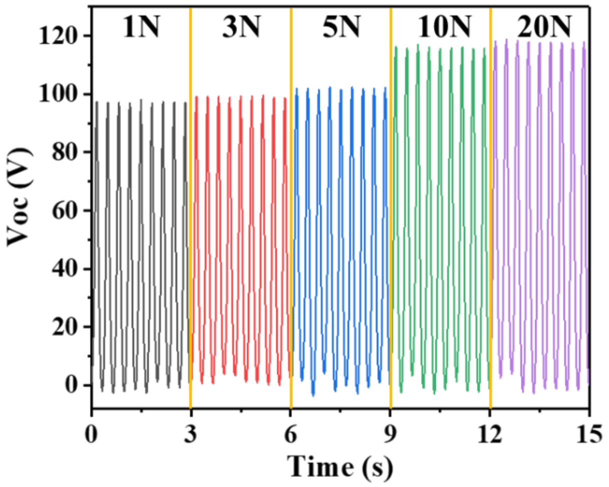

Fig. S10 V_{OC} of the STENG with various applying pressure (fixed frequency of 5 Hz)

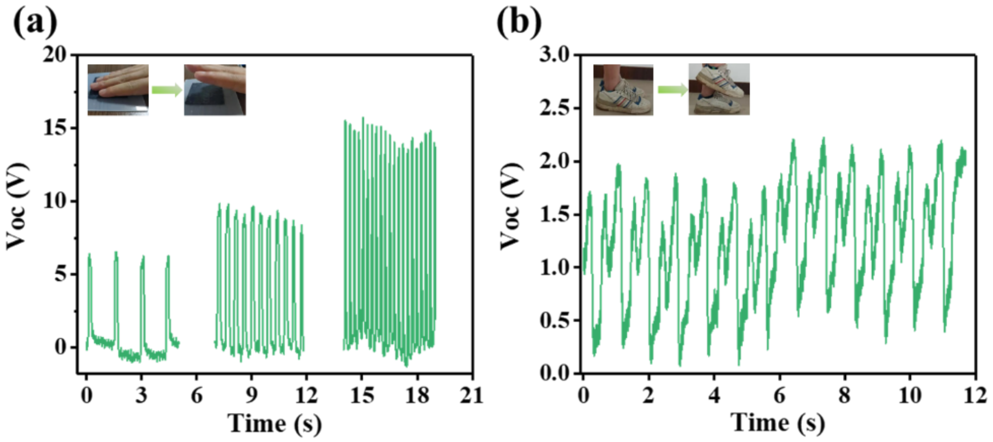

Fig. S11 Corresponding Voc of the MxAgy-based STENG to monitor **a** finger bending of different frequencies and **b** regular steps

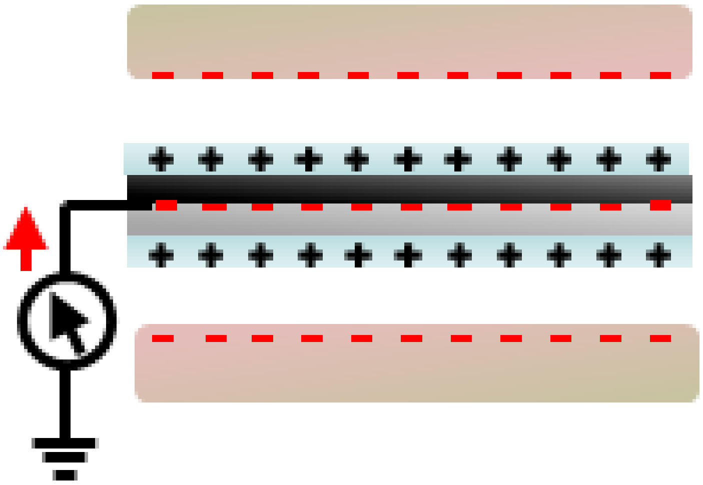

Fig. S12 Electrical output mechanism of double triboelectrification layers
